# A first exploration of a social identity perspective on youth rugby talent development in Ireland

**DOI:** 10.3389/fspor.2026.1715525

**Published:** 2026-03-13

**Authors:** Emma Burrows, Deirdre Lyons, Tarli Young

**Affiliations:** 1 Rugby Players Ireland, Dublin, Ireland; 2 University College Dublin, Dublin, Ireland; 3 School of Psychology, The University of Queensland, Brisbane, Queensland, Australia

**Keywords:** athletic identity, rugby, social identity, talent development, well-being

## Abstract

This study uses a social identity perspective to explore the possible relationships between athletic identity, social identities in sport, and players’ life satisfaction and confidence in managing their social groups. We report cross-sectional data from 27 of the 64 early-career academy-contracted rugby players in Ireland in 2025. Exploratory analyses indicate that players endorsed stronger identification with their sport and their club than with a singular athletic identity. Importantly, life satisfaction was positively associated with their confidence in managing multiple social groups but not the strength of their athletic identity. This is consistent with the social identity model of identity change (SIMIC), which posits that belonging to multiple social groups and managing these identities effectively supports well-being during transitions, such as progressing into professional sport. This perspective suggests that fostering early-career athletes’ confidence to balance and maintain diverse social identities including family, education, and friendships, may support their well-being.

## 1 Introduction

A rugby career is made of many transitions such as the entry to professional status, the promotion to international squads, changing clubs, and retirement. Coping well with these transitions is an integral part of a player's career as difficulty coping with career transitions may contribute to negative outcomes (e.g., premature dropout and poor adjustment) and, for some athletes, is associated with maladaptive coping behaviors such as harmful alcohol and drug use (1, 2).

Entry into professional rugby in Ireland is undertaken via the provincial Academy system. Typically, players contracted to a provincial academy are between 18 and 20 years of age. Before being offered a professional academy contract they may have played for their school or club, been selected to represent Ireland at under 19 or under 20s competitions or trained with a pre-academy cohort in one of the provinces' centers of excellence. Given their age profile they are also likely to be experiencing educational and psychosocial transitions. A body of research discusses how a player's relative ability to adapt to these transitions is largely influenced by their athletic identity (3).

Athletic identity refers to the degree to which individuals define themselves primarily and exclusively as athletes (4). A strong athletic identity can enhance motivation and performance during an athlete's career, but the loss of this identity (e.g., when leaving sport) can undermine adjustment and well-being (1, 5). Increasingly, the psychology literature on transitions and coping has also suggested that an individual's social group membership is a key resource for support in these transitions. More recently, researchers have begun to consider the role of social identity in coping with the transitions within the professional sport career (6). For example, sport and club identities capture players' sense of belonging to rugby as a sport and to their specific club. However, there has been very little emphasis on measuring how early-career rugby players identify with the sport, their team and how any of these factors affect their life satisfaction. The social identity approach to health offers a lens to examine the effects of these social groups during players' transitions into the professional game (6).

### 1.1 The role of social identity in athlete transition

The social identity approach to health explains how social group dynamics influence health, and well-being (7). This approach applies both social identity theory (8), and self-categorization theory (9). Social identity theory suggests that identity is shaped not only by individual traits, but also by memberships of social groups. In other words, people can see themselves in terms of both “I” and “We” (10). Most people possess multiple social identities as they belong to various groups. For example, a young athlete considering a professional rugby contract may belong to a family, friendship group, religion, school, other sports team, rugby club team, and underage national team. Self-categorization theory suggests that when people identify with meaningful groups, the group norms influence their thoughts, behaviors, and feelings. In a sporting setting this process of self-categorization into the team identity helps teams to work cooperatively towards a common goal (11). Investing in social identity processes can therefore benefit both sports organizations and athletes. Athletes who strongly associate with their sport can gain psychological resources such as social support, self-esteem, and a sense of purpose (7, 12). However, these resources gained from the sport and club social identities can be lost during periods of injury or retirement, leading to poor well-being outcomes, making identity diversification critical to longer term well-being. For example, recent work by Haslam et al. (13) explored the impact of social identity loss in athletic retirement through the social identity approach to health. This work found that losing one's sporting identity can significantly undermine well-being by limiting access to psychological resources offered by group membership. This impact can be explained by the social identity model of identity change.

### 1.2 Social identity model of identity change: navigating life transitions in sport

The social identity model of identity change provides a general framework for understanding why identity disruptions during major life changes pose a particular threat to people's well-being*.* In a sporting context, SIMIC provides valuable insights into how athletes navigate life transitions such as entry into, and retirement from, sport and the associated shifts in identity that come with these transitions (7, 12, 13). According to this model, four key processes help facilitate successful adaptation to career transitions. First, belonging to multiple groups before a transition ensures access to diverse support networks, which can buffer the impact of identity change and loss. Second, maintaining connections with pre-existing groups provides a sense of continuity, offering stability and psychological resources during periods of change. Third, gaining new social identities post-transition helps individuals offset identity loss and find renewed meaning. Finally, the compatibility between different identities plays a crucial role in well-being, as transitions are often smoother when new roles feel compatible with previous group affiliations (11, 13, 14). For example, a rugby player entering the professional game may benefit from retaining their membership of other valued groups such as family, friends, education, and sports club. The SIMIC model was applied during longitudinal research with junior elite cricketers which demonstrated that having more group memberships before and after entering high-performance pathways predicts better coach-rated performance, life satisfaction, positive affect, and self-esteem (27). In a rugby setting, these results are relevant for the onboarding and integration of early-career rugby players into the professional environment.

### 1.3 The current study: applying SIMIC in the Irish rugby context

In Ireland, the average professional rugby career lasts 8 years (15) and coaches and backroom teams are under pressure to make good selection decisions and to integrate players as quickly as possible. Due to the short-lived careers Irish rugby has dedicated support systems in place to help players manage their career transitions. Each provincial professional team in Ireland has a dedicated Player Development Manager (PDM) based at the club who promotes player well-being and works individually with players on their development “off the pitch”. Player development managers are invested in a player’s long-term physical, mental and social health, which are important for both players' well-being and team performance (16). Services like this exist in professional rugby environments across the world. In the Irish context multiple stakeholders such as the governing body, the IRFU, the four provincial organizations, and the player's association (Rugby Players Ireland), work together to provide access to off-field personal development.

Despite robust player-care systems and extensive work on athletic identity, comparatively little attention has been paid to rugby players' social identities; particularly how their sport, club, and broader social identities relate to their well-being during early career transitions. Social identity approaches have been successfully applied to retirement transitions, with athletes emphasizing that support should begin well before retirement, by helping them maintain connections beyond sport throughout their careers (13, 14). The current study is the first to apply a social identity lens to the transition of early-career rugby players into professional sport. Specifically, we survey players to examine different forms of identity (athletic identity, sport identity, club identity) and players' self-efficacy in managing their social identities. We also explore how these outcomes relate to life satisfaction. Life satisfaction captures young players' overall sense of how their lives are going and provides a core indicator of well-being that has been widely used in research on sporting transitions (11, 13).

## 2 Methods

### 2.1 Procedure

Year 1 and 2 contracted professional players were asked to complete a short survey online. All participants provided informed consent prior to participation. The survey included measures on identity and life satisfaction (described below) and collected basic demographic data. The survey was distributed to players via a WhatsApp group on Survey Monkey. Data was collected in August 2025, which is the pre-season training block, approximately 6 weeks into the contract of Year 1 players. All procedures received approval from the University of Queensland Research Ethics and Integrity committee (2020/HE002920).

### 2.2 Participants

The sample comprised all 64 male players who held a Year 1 or 2 academy contract with one of the four Irish provincial teams in 2025. These academy-contracted players are best described as falling between Tier 3 (highly trained/national level), and Tier 4 (Elite/International level) of McKay et al. (17) framework given their training volumes and position within a professional pathway. Twenty-seven players completed the survey (response rate 42%). Participants were aged 18–21 years (*M* = 19.81). Most (48%) reported being single and never married, 44% were in a relationship but not living together, and 8% preferred not to answer. All had completed school leaving exams and 78% were currently studying for a higher qualification.

### 2.3 Measures

#### 2.3.1 Sport identity

Sport identity was measured using four items adapted from (18). Participants responded to statements including “I identify with members of my sport” using a 5-point Likert scale ranging from 1 (do not agree at all) to 5 (agree completely). Scores were averaged across items, with higher scores reflecting greater sport identification. Internal consistency in the present sample was good (*α* = .87).

#### 2.3.2 Athletic identity

Athletic identity was assessed using the 7-item Athletic Identity Measurement Scale (19). The scale includes items such as “I consider myself an athlete” and “Sport is the most important part of my life,” rated on a 7-point Likert scale from 1 (strongly disagree) to 7 (strongly agree). Item scores were summed to produce a total athletic identity score, with higher scores indicating stronger identification with the athlete role. In this small sample, internal consistency was poor (*α* = .41). Item inspection suggested restricted variance for the sports goals item (“I have many goals related to sport”; ceiling effects) and weak/negative associations involving the negative affect item (“I would be very depressed if I were injured and could not compete in sport”). Given evidence consistent with multidimensionality in this sample, results involving athletic identity should be interpreted cautiously.

#### 2.3.3 Club identity

Club identification was assessed by five items adapted from Boen et al. (20) and De Backer et al. (21). Sample items include “Being a member of my club is very important for me.” These were rated on a 5-point Likert scale ranging from 1 (do not agree at all) to 5 (agree completely). Scores were averaged across items, with higher scores reflecting greater team connection. Internal consistency was acceptable (*α* = .78).

#### 2.3.4 Social group management (SEMSI)

The SEMSI scale (22) consists of six items that measure confidence in managing social group memberships. Items such as “I am confident that I can maintain strong connections with my groups during difficult times” were rated on a 5-point scale from 1 (strongly disagree) to 5 (strongly agree). Scores were averaged across items, with higher scores reflecting greater confidence. Internal consistency was good (*α* = .87).

#### 2.3.5 Satisfaction with life

Life satisfaction was measured by a single item “All things considered, how satisfied are you with life as a whole these days?” Responses ranged from 0 (completely dissatisfied) to 10 (completely satisfied). The raw score was used (23, 24).

## 3 Exploratory results

Given the small sample size and exploratory aims, analyses focused on descriptive statistics (means, standard deviations) and Pearson correlations among all variables. Analyses were treated as exploratory and are only powered to detect large associations. The mean scores and correlations of all survey items are presented below in Table 1.

**Table 1:** Descriptive statistics and intercorrelations of T1 variables (*N* = 27).

Variable	M	SD	1	2	3	4	5
1. Sport Identity	6.32	0.92	—				
2. Athletic Identity	5.65	0.59	.48*	—			
			[0.13, 0.73]				
3. Club Identity	6.28	0.77	.41*	0.23	—		
			[0.04, 0.69]	[−0.17, 0.56]			
4. Self-efficacy in Managing Social Identities (SEMSI)	5.60	0.94	0.38	–.01	.56**	—	
			[−0.01, 0.66]	[−0.39, 0.37]	[0.23, 0.78]		
5. Life Satisfaction	5.63	0.81	0.06	0.02	0.28	.41*	—
			[−0.33, 0.43]	[−0.36, 0.40]	[−0.11, 0.60]	[0.04, 0.69]	

Correlations are Pearson’s *r* (two-tailed) and appear below the diagonal. *p < .05, **p < .01. Values in brackets represent the lower and upper bounds of the 95% confidence interval. All measures have been scaled to a 1-to-7-point scale to allow comparisons across scales.

On average, players reported high levels of identification with rugby as a sport and with their club, and somewhat lower levels of athletic identity. Regarding correlations, there was a significant association between athletes' sport identity and both their athletic and club identity. Life satisfaction correlated with self-efficacy in managing social identities. This indicates that players’ ability to manage their own social groups was associated with their well-being. Sports-based identities did not have the same association with life satisfaction however, with only 27 participants, this analysis is powered only to identify large correlations, and there may be smaller associations that would be visible with a larger sample.

## 4 Discussion

Early professional sport is not a single transition but a cascade of identity transitions that can shape both well-being and performance. To examine how social identity processes operate at this entry point, we surveyed Irish academy players' sport-, club-, and athlete-based identities and their self-efficacy for managing social identities and examined how these related to life satisfaction. As newcomers to professional rugby, these players already show a strong sense of belonging both to rugby as a sport and to their own club. This pattern suggests that Irish clubs may be effective in fostering early connection and identification among new academy recruits. By comparison, the relatively moderate levels of athletic identity, combined with stronger sport and club identities, may reflect their position at the start of their professional journey, when belonging to rugby and their club is salient but an exclusive “athlete-only” identity has not yet fully consolidated. When it comes to well-being, the strength of any single sport-based identity was not associated with player's life satisfaction, although the small sample size may have obscured smaller correlations. Meanwhile, life satisfaction was associated with players' confidence in managing the different social groups in their lives. The capacity to maintain, build, and manage multiple identities, rather than simply embracing one, seems to be important to players' well-being. This pattern is consistent with recent social identity analyses of athlete well-being, which emphasize that multiple, well-managed group memberships act as psychological resources that support flourishing, particularly in elite and high-performance sport (6). Interestingly, most of the participants in the current study were engaged in higher education in addition to rugby, which may support their membership in multiple valued groups beyond rugby and their long-term career preparation.

Taken together, these findings suggest that in the early stages of a professional rugby career it is players' confidence in managing social identities, not merely the intensity of a budding athlete identity, may provide psychological benefit. Early-career players may therefore gain more well-being by nurturing their ability to manage their social worlds than by trying to fast-track a singular “athlete” identity.

## 5 Implications

While social identity approaches have been applied to athlete retirement (13, 14), the current results emphasize the relevance of such approaches to the early stages of athlete careers. Specifically, the significant association between players' self-efficacy in managing social identities and their life satisfaction supports the SIMIC model by highlighting the importance of being able to manage multiple social identities.

At a practical level, for player-care systems and onboarding programs, the current findings suggest that supporting young athletes to maintain and manage multiple identities across family, education, friends, and clubs may offer life satisfaction benefits worth pursuing. Social group memberships can support performance outcomes such as heart rate recovery (25) and task persistence (26), suggesting implications for both well-being and talent development. This aligns with recent recommendations that athlete-support systems deliberately monitor and foster athletes' group memberships, using tools such as social identity mapping, to protect well-being in elite sport (6). The above results and the SIMIC model offer an insight into how to maximize the successful integration of a player into the professional environment. Meanwhile, efforts to help players to maintain, gain, and manage social identities (such as the More Than Sport intervention), may help protect them from the identity loss and subsequent decline in well-being that can come with life transitions, including their transition into professional sport and the inevitable transition that comes with retirement from professional sport.

## 6 Strengths and limitations

To our knowledge, this is the first study to examine social identity patterns and life satisfaction in early-career professional rugby players, offering baseline insights to guide future longitudinal and intervention research. In doing so, this exploratory study begins to address recent calls to apply the social identity approach to athlete well-being during career transitions. By quantifying multiple sport-related identities and confidence in managing group memberships in academy players, we can see links to life satisfaction at the point of entry into professional rugby (6).

Several limitations should be noted, however. The sample was small (*N* = 27) and limits the visibility of small effects, while the specialized population of Irish rugby academy players limits generalizability. The data are cross-sectional exploratory observations, so no causal conclusions can be drawn. In addition, the athletic identity scale showed poor internal consistency in this sample, which may attenuate observed associations with life satisfaction and suggests that findings involving this construct should be interpreted cautiously.

## 7 Conclusion

Understanding social identity processes is crucial in youth rugby talent development, both for well-being and performance. The SIMIC model offers a compelling framework for navigating these changes, emphasizing the importance of multiple group memberships, identity maintenance and gain, alongside identity compatibility. By integrating these insights, organizations can better support young athletes, helping them develop beyond their sport, and fostering resilience for life after competition.

## Data Availability

The raw data supporting the conclusions of this article will be made available by the authors, without undue reservation.
